# Revealing a Brain Network Endophenotype in Families with Idiopathic Generalised Epilepsy

**DOI:** 10.1371/journal.pone.0110136

**Published:** 2014-10-10

**Authors:** Fahmida A. Chowdhury, Wessel Woldman, Thomas H. B. FitzGerald, Robert D. C. Elwes, Lina Nashef, John R. Terry, Mark P. Richardson

**Affiliations:** Institute of Psychiatry, Psychology and Neuroscience, King's College London, London, United Kingdom; Centre for Epilepsy, King's College Hospital, London, United Kingdom; College of Engineering, Mathematics and Physical Sciences, University of Exeter, Exeter, United Kingdom; Wellcome Trust Centre for Neuroimaging, UCL, London, United Kingdom; Wake Forest School of Medicine, United States of America

## Abstract

Idiopathic generalised epilepsy (IGE) has a genetic basis. The mechanism of seizure expression is not fully known, but is assumed to involve large-scale brain networks. We hypothesised that abnormal brain network properties would be detected using EEG in patients with IGE, and would be manifest as a familial endophenotype in their unaffected first-degree relatives. We studied 117 participants: 35 patients with IGE, 42 unaffected first-degree relatives, and 40 normal controls, using scalp EEG. Graph theory was used to describe brain network topology in five frequency bands for each subject. Frequency bands were chosen based on a published Spectral Factor Analysis study which demonstrated these bands to be optimally robust and independent. Groups were compared, using Bonferroni correction to account for nonindependent measures and multiple groups. Degree distribution variance was greater in patients and relatives than controls in the 6–9 Hz band (p = 0.0005, p = 0.0009 respectively). Mean degree was greater in patients than healthy controls in the 6–9 Hz band (p = 0.0064). Clustering coefficient was higher in patients and relatives than controls in the 6–9 Hz band (p = 0.0025, p = 0.0013). Characteristic path length did not differ between groups. No differences were found between patients and unaffected relatives. These findings suggest brain network topology differs between patients with IGE and normal controls, and that some of these network measures show similar deviations in patients and in unaffected relatives who do not have epilepsy. This suggests brain network topology may be an inherited endophenotype of IGE, present in unaffected relatives who do not have epilepsy, as well as in affected patients. We propose that abnormal brain network topology may be an endophenotype of IGE, though not in itself sufficient to cause epilepsy.

## Introduction

Idiopathic generalised epilepsy (IGE) comprises a group of clinical syndromes which account for 15–20% of all epilepsies [1]. Although the classification scheme for the epilepsies is evolving, the concept of IGE remains robust, consisting of a set of epilepsy disorders characterised by specific well-recognised generalised seizure types. Although IGE may very rarely be a monogenic disorder in a few families [2], typically it has a complex inheritance suggesting susceptibility is associated with multiple genes [3].

Generalised spike-wave (GSW) seen in EEG is a hallmark of IGE, and reflects abnormal hypersynchronous electrical activity within brain networks. There is at present much interest concerning the structural and functional nature of brain networks in which seizures arise [4] and how these factors give rise to specific seizure types or epilepsy syndromes. The complexity of the brain makes it challenging to study, but a well-developed approach to characterising complex networks, graph theory, has recently had a substantial impact on the investigation of data relating to brain networks [5]. Graph theory enables local and global characteristics of network connectivity to be computed and compared between subjects. Brain networks can be inferred from EEG by examining the patterns of association between EEG signals (correlation, synchronisation etc), based on the ability of EEG to capture information about multiple brain sources of activity. It is assumed that neuronal activity in distributed brain networks is reflected in multiple sources of independent activity detectable in scalp EEG, and that examining interactions between the signals obtained by different EEG electrodes is a reasonable proxy for examining interactions between the underlying sources which constitute the brain network. Graph theory can be used to summarize structural topological features of brain networks; these structural properties may have a key influence on the dynamics which the network can generate [4]. Abnormality of brain dynamics is evident in epilepsy as the paroxysmal occurrence of seizures, therefore it is logical to propose that these abnormal dynamics may be dependent on abnormal network topology. The aim of this study is to use graph theory applied to EEG to explore the hypothesis that abnormal properties of brain networks are a component of the inherited phenotype in IGE.

Investigations of the complex genetics of brain disorder have in some instances made important progress through investigating endophenotypes, heritable traits with a simpler genetic basis than the full disorder, which may be present in family members who do not have the disease [6]. Measures of network topology have been suggested as potential endophenotypes [5]. It is noteworthy that some basic EEG-derived network metrics obtained using graph theory, particularly clustering coefficient and average path length, show high heritability in healthy subjects, especially in the alpha frequency band [7], [8]. Studies of the maturation of brain networks in children [9] suggest that normal development is characterised by a gradual alteration of the balance between the strength of local connectivity, presumably reflecting cortical localisation of function, and the strength of long-range connections which presumably reflects the functional integration between localised regions required for normal brain function. From a graph theoretic perspective, this balance is reflected in the small-world index. Given that IGE may often have onset in childhood and remit with maturation, we specifically hypothesise that brain networks in people with IGE and their relatives will show altered network properties compared to healthy controls, and that this may have a basis in aberrant development.

Interpretation of EEG in a clinical setting typically uses five broad frequency bands defined according to prominent features visible to an expert observer. A recent literature has sought to establish the frequency bands in which EEG oscillatory activity is maximally independent, hypothesising that such maximally-independent bands may represent different neurobiological generators, and may be optimally sensitive to differences between subjects or experimental manipulations. Although the conventional clinical EEG frequency bands relate to qualitative features seen in the EEG, it is not necessarily the case that these conventional bands optimally reflect the underlying generators. Furthermore, given that brain network features in the alpha band may show evidence of heritability [7], [8], and that antiepileptic drug treatment my alter peak alpha frequency [10], we particularly focus on the alpha range through dividing into sub-bands. Here, we adopt the frequency bands defined by Spectral Factor Analysis (SFA) in two independent datasets of resting EEG activity [11], in which these bands were shown to be extremely robust to a range of methods used to determine the bands, artefact rejection schemes and scalp electrode positions.

## Materials and Methods

### Recruitment and selection of participants

Subjects with IGE were identified from five hospitals in London and outlying regions, and were a consecutive series that met the inclusion and exclusion criteria and were able to participate. Inclusion criteria for patients were age>18 years old, a diagnosis of IGE, and ≥2 family members with epilepsy according to self-report. Twenty-eight families were recruited; in 16 families the reported presence of epilepsy in more than one family member was confirmed by us from history and investigation; in the other 12 families, the reportedly affected family members were not available for assessment. In addition to the affected probands, clinically unaffected first degree relatives were recruited from the 28 families. These unaffected relatives were interviewed in detail by a neurologist (FAC) and had no evidence of symptomatic seizures from detailed history. Furthermore, in addition to the EEG study carried out as part of this investigation, all unaffected relatives underwent diagnostic MRI which was in all cases normal. Healthy participants with no personal or family history of neurological or psychiatric diseases were recruited via a local research participant database. Participants were excluded if they had any other neuropsychiatric condition or a full scale IQ (FSIQ) <70. Ethical approval was obtained from King's College Hospital Research Ethics Committee (08/H0808/157). Written informed consent was obtained from all participants. We recently reported the neuropsychometric findings in this cohort of patients, relatives and controls [12].

### EEG acquisition

Conventional 10–20 scalp EEG was collected using a NicoletOne system (Viasys Healthcare, San Diego, California, USA), 19 channels, sampling rate 256 Hz, bandpass filtered 0.3–70 Hz. EEG was carried out using the same system in the same recording room, undertaken by the same EEG technologist using conventional measurement techniques to determine electrode positions. Collection of subjects from the different groups was interleaved over the duration of the study. Ten minutes of awake EEG in all participants and 40 minutes of sleep was obtained where possible. Where specific consent was obtained, hyperventilation and photic stimulation were carried out. Here we examined only the awake EEG.

### Conventional expert EEG analysis

The EEGs were reviewed independently by two reviewers (FC and RE). The following features were noted: presence of GSW; focal abnormalities including spikes, sharp waves and slow waves; response to photic stimulation; and normal variants.

### Quantitative EEG analysis

EEG data was referenced to the channel average. A single 20 s epoch was selected which included continuous dominant background rhythm with eyes closed, without any artefacts, epileptiform abnormalities or patterns indicating drowsiness or arousal. Epoch selection for analysis was carried out by one investigator (TF) who was blinded to subject group. These EEG epochs were used for all the subsequent analysis methods described below. Our analyses used 5 frequency bands defined from previous literature applying SFA to resting EEG: 1–5 Hz, 6–9 Hz, 10–11 Hz, 12–19 Hz and 21–70 Hz. Although different from the conventional clinical EEG frequency bands, the bands we used here were shown to be extremely robust to a range of methods used to identify the maximally independent bands, artefact rejection schemes and scalp electrode positions [11].

Analyses were performed using a combination of EEGlab toolbox [13], the Brain Connectivity Toolbox [14], in addition to our own custom Matlab (Mathworks, Natick, Massachusetts, USA) scripts for band-pass filtering the EEG data to optimise the rectangular drop-off at the boundary between frequency bands.

### Construction of weighted undirected graphs

The Hilbert transform was applied to the band-pass filtered EEG to generate instantaneous phase and amplitude estimates. For each electrode pair and each frequency band, we calculated the phase-locking factor (PLF) [15], a value between 0 and 1 reflecting the strength of synchronous activity between each pair. We assumed that each electrode is represented by a vertex in a graph with edge strength between vertices determined by the relevant PLF. All PLF analyses were carried out using custom scripts implemented in Matlab (available from authors on request). Note that we therefore construct weighted graphs, with each edge taking the value of the corresponding PLF.

### Degree distribution, clustering coefficient, characteristic path length

For each individual, we characterise the degree distribution by establishing the strength of each vertex through summing the PLF values associated with the edges connected to that vertex and then using the mean and variance of these vertex strengths, denoted by *K* and *D* respectively. The clustering coefficient *C* indexes the tendency of a network to form local clusters; the path length *L* is a measure of how well the nodes of the network are interconnected [16]. *C* and *L* are sensitive to changes in network degree distribution [16], [17]. To control for this, we calculated normalised metrics 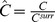 and 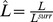 where *C^surr^* and *L^surr^* are the mean clustering coefficient and characteristic path length of a distribution of 500 surrogate random networks [16], [17]. We calculated 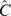 and 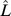 for each subject for each frequency band network. All network topology analyses were carried out using the Brain Connectivity Toolbox [14].

### Statistical testing

To explore differences in the proportions of each group showing qualitative EEG abnormalities we used a Chi-squared test with significance threshold of p = 0.05 two-tailed, Bonferroni-corrected for three between-group comparisons.

Prior to testing, all quantitative measures were tested for normality and a non-normal distribution was observed. Thus a non-parametric Kruskal-Wallis test was used to examine for effects in each measure across the three groups and five frequency bands; results were declared significant at p<0.05 two-tailed, Bonferroni corrected for five frequency bands. Where the Kruskall-Wallis test was significant, we investigated further using Mann-Whitney tests to compare between pairs of groups for each frequency band. Results were declared significant when p<0.05 after Bonferroni correction for three between-group comparisons.

## Results

We studied 117 participants: 40 normal controls (20 female, mean age 30.7 yrs), 35 patients with IGE (21 female, mean age 34.4 yrs), and 42 unaffected first-degree relatives of patients with IGE (19 female, mean age 36.0 yrs). The age and gender distributions of the groups were not significantly different (all p>0.05 uncorrected). Clinical details of the patients who participated in the study are presented in Table 1. Thirteen patients and 8 relatives refused photic stimulation because of the risk of provoking a seizure.

**Table 1:** Clinical characteristics of the patients.

Gender	Age	Syndrome	Age of onset (years)	Seizures and frequency	Time since last seizure	Medications (total daily dose mg)	EEG	MRI
M	26	GTCS	5	GTCS 1/month	2 weeks	Sodium Valproate 1600, Topiramate 200, Lamotrigine 100	GSW	Normal
M	25	GTCS	11	GTCS 3/month	3 weeks	Sodium Valproate 300	GSW	Normal
F	45	GTCS	2	SF	36 years	(none)	Normal	N/A
M	31	GTCS	8	GTCS 6/year	1 month	Sodium Valproate 2000, Zonisamide 250, Levetiracetam 500, Lamotrigine 100	GSW, Ph+	N/A
F	18	JAE	7	GTCS 1/month, Abs SF	1 week	Ethosuximide 250, Lamotrigine 600	GSW	Normal
F	20	GTCS	0.5	SF	9 years	(none)	Normal	N/A
M	49	GTCS	26	SF	1 year	(none)	GSW	Normal
F	21	JAE	10	SF	4 years	Lamotrigine 400, Ethosuximide 500	GSW	Normal
F	20	JME	13	MJ weekly, GTCS SF	1 week	Sodium Valproate 1000	GSW	Normal
M	59	JME	14	SF	10 years	(none)	GSW	N/A
F	19	GTCS	15	GTCS 4/year	3 months	Levetiracetam 2000	GSW	Normal
F	28	Unclassified	20	SF	7 years	Carbamazepine 200	Normal	Normal
F	23	CAE	8	SF	6 years	Sodium Valproate 800, Lamotrigine 25	GSW	Normal
M	48	JME	17	SF	5 years	Sodium Valproate 1500, Topiramate 200, Carbamazepine 600	GSW, PSW	N/A
F	32	CAE	4	GTCS SF, Abs weekly	1 weeks	(none)	GSW	N/A
M	30	Unclassified	11	SF	3 years	(none)	Normal	N/A
F	28	JME	15	SF	13 years	Sodium Valproate 1400	PSW	N/A
F	41	JME	11	GTCS rare, MJ weekly	1 week	Levetiracetam 1000, Lamotrigine 500, Zonisamide 200	GSW, PSW	N/A
M	45	CAE	3	SF	2 years	Sodium Valproate 1400, Levetiracetam 2000	GSW	N/A
M	31	Unclassified	8	SF	10 years	Sodium Valproate 400	Normal	Normal
M	27	Unclassified	16	SF	10 years	Carbamazepine 1200	Normal	N/A
F	39	GTCS	22	SF	10 years	Carbamazepine 200	GSW	Normal
M	28	CAE	4	SF	5 years	Sodium Valproate 600, Levetiracetam 750, Lamotrigine 250	GSW	N/A
F	18	JME	15	MJ SF, GTCS 1/month	4 months	Levetiracetam 1000	GSW	N/A
F	36	GTCS	21	GTCS 2/year	2 months	Levetiracetam 1750	GSW, Ph+	Normal
F	43	CAE	7	SF	10 years	(none)	GSW	N/A
M	28	GTCS	8	SF	1 year	Sodium Valproate 400	GSW	N/A
F	53	GTCS	3	GTCS SF, Abs daily	1 day	(none)	GSW, Ph+	Normal
F	33	JAE	12	GTCS 3/year	4 months	Topiramate 400	GSW, Ph+	Normal
F	55	GTCS	16	SF	25 years	(none)	Normal	N/A
M	26	CAE	5	SF	8 years	(none)	GSW	N/A
F	47	JAE	11	Abs daily, GTCS SF	1 day	Levetiracetam 2000	PSW	Normal
M	25	JME	14	GTCS 5/year, MJ weekly	1 week	Valproate	GSW	Normal
F	20	JME	15	MJ 2/month	2 weeks	Lamotrigine 400, Levetiracetam 1500	GSW	Normal
F	21	Absences with eyelid myoclonia	6	Abs daily, MJ weekly	1 day	Lamotrigine 500	PSW	N/A

CAE childhood absence epilepsy, GTCS generalised tonic clonic seizures only, JAE juvenile absence epilepsy, JME juvenile myoclonic epilepsy, MJ myoclonic jerks, Abs absences, Ph + Photosensitivity; GSW generalised spike and wave, PSW polyspike and wave; SF Seizure Free; N/a not available.

### Qualitative Analysis

Patients were more likely to have generalised epileptiform discharges compared with relatives and controls (17/35 patients, 2/42 relatives, 0/40 controls; chi-squared with Fisher's exact test, one-sided p<0.0001 Bonferroni corrected in both instances), but there was no significant difference in the proportion of relatives with generalised epileptiform discharges compared with normal controls (p = 0.27 uncorrected). There were no significant differences between any pair of groups in the proportions of subjects with focal discharges, positive photoparoxysmal response or normal variants.

### Graph theoretic metrics (Figure 1, Table 2)

**Figure 1:**
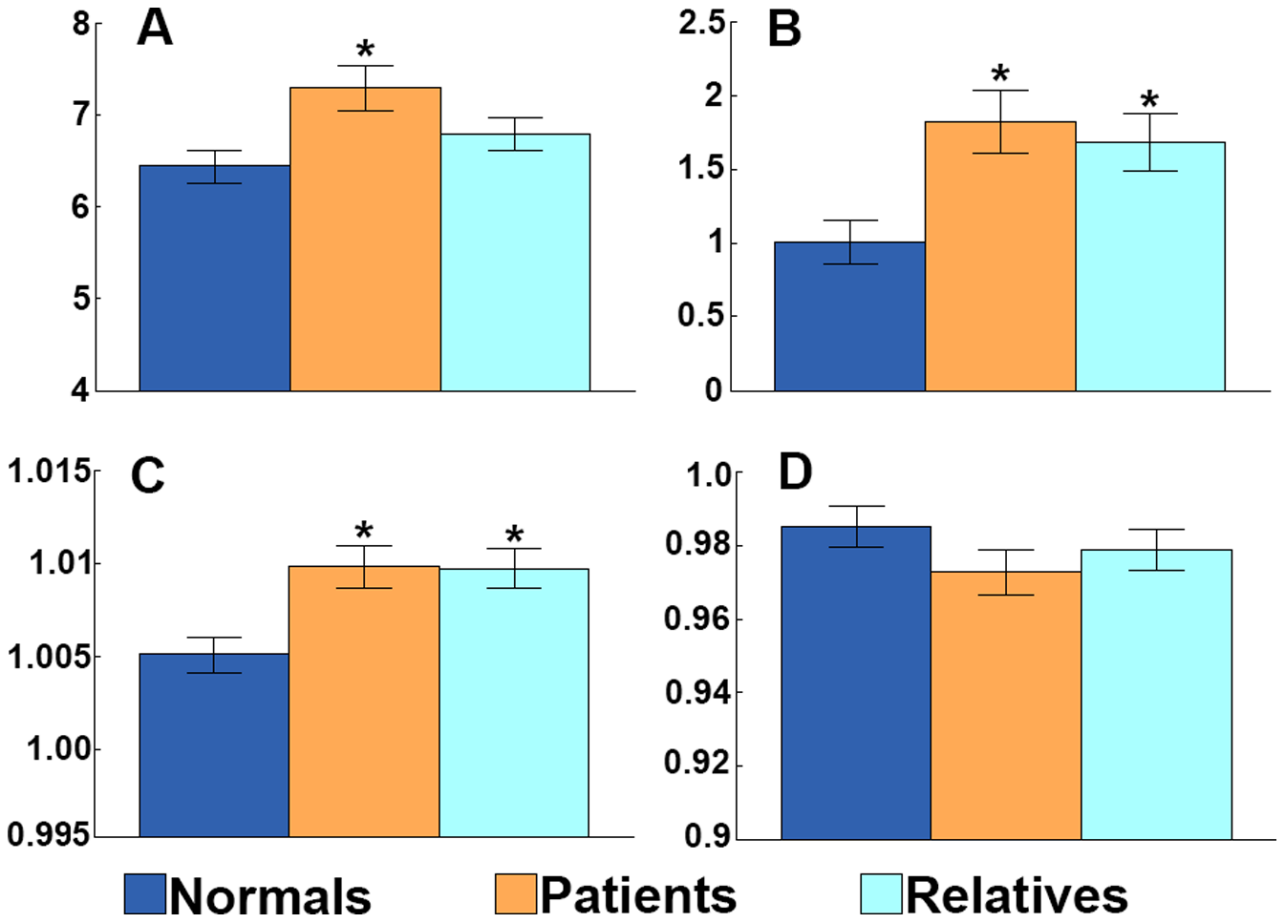
An abnormal EEG network topology is an endophenotype of IGE, present in patients and first-degree relatives. Group means +/− standard error of the mean are shown for: (A) mean degree *K*, (B) mean degree variance *D*, (C) clustering coefficient 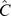, and (D) normalised path length 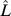, in the 6–9 Hz band. Normal controls (dark blue), patients with IGE (orange), and first-degree relatives of patients with IGE (light blue). *  =  p<0.05 Bonferroni corrected compared with normal controls.

**Table 2:** Summary of effects found comparing three groups (normal controls, patients, relatives).

Measure	Comparison	1–5 Hz		6–9 Hz		10–11 Hz		12–19 Hz		21–70 Hz	
		Uncorr.	Bonferroni corrected	Uncorr.	Bonferroni corrected	Uncorr.	Bonferroni corrected	Uncorr.	Bonferroni corrected	Uncorr.	Bonferroni corrected
**Mean degree**	difference between groups	0.9513		0.0013	0.0064	0.9321		0.5501		0.5435	
**Mean degree**	normals vs patients			0.0003	0.0008						
**Mean degree**	normals vs relatives			0.0514							
**Mean degree**	relatives vs patients			0.0718							
**Mean degree variance**	difference between groups	0.8795		0.0001	0.0005	0.8533		0.6280		0.0441	0.2206.
**Mean degree variance**	normals vs patients			0.0002	0.0005						
**Mean degree variance**	normals vs relatives			0.0003	0.0009						
**Mean degree variance**	relatives vs patients			0.5947							
**Clustering coefficient**	difference between groups	0.9003		0.0004	0.0018	0.7291		0.5370		0.1315	
**Clustering coefficient**	normals vs patients			0.0008	0.0025						
**Clustering coefficient**	normals vs relatives			0.0004	0.0013						
**Clustering coefficient**	relatives vs patients			0.8780							
**Characteristic path length**	difference between groups	0.5920		0.0814		0.4343		0.3798		0.8177	
**Characteristic path length**	normals vs patients										
**Characteristic path length**	normals vs relatives										
**Characteristic path length**	relatives vs patients										

For details of Bonferroni correction see Methods. Uncorr  =  uncorrected.

Mean degree (*K*) differed between the groups only in the 6–9 Hz band (Kruskall-Wallis p = 0.0064, Bonferroni corrected for five frequency bands). Subsequent comparison of group pairs revealed that *K* was higher in the patients than normal controls (Mann-Whitney p = 0.0008, Bonferroni corrected for three between-group comparisons); in relatives, K was higher than healthy controls and lower than patients but did not differ significantly from either group. Degree distribution variance (*D*) showed a difference between the three groups only in the 6–9 Hz band (p = 0.0005, Bonferroni corrected for five frequency bands). Examining paired comparisons between groups, *D* was higher in patients and relatives than in normals in this band (p = 0.0005 and p = 0.0009 respectively, Bonferroni corrected). Clustering coefficient (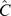) differed between the three groups only in the 6–9 Hz band (p = 0.0018, Bonferroni corrected). 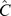 was greater in the patients and relatives than in normal controls (p = 0.0025 and p = 0.0013 respectively, both Bonferroni corrected). There were no differences between groups for 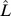. There were no other significant differences or trends between groups in any other frequency band, comparing controls, patients and relatives. In particular, there were no differences between patient and relative groups in any frequency band for any measure.

## Discussion

In this study we show that brain network topology, as inferred from scalp EEG, differs between normal subjects and patients with IGE. Moreover, we show that brain network topology differs between normal subjects and unaffected first-degree relatives of people with IGE – and that unaffected relatives and patients have similar networks. Although it is conceivable that EEG network features in the patients may differ from normal subjects as a result of antiepileptic drug treatment, the unaffected relatives were not taking medication. We conclude that brain network topology may be a component of an inherited endophenotype of IGE, and not dependent on medication effects.

We have previously reviewed in detail the literature describing brain networks in epilepsy using a wide range of approaches, not only graph theory [18]. We are not aware of prior literature examining brain network data from unaffected relatives of patients with IGE; however there is a small published literature examining brain networks of patients with IGE, using graph theory methods, in comparison with normal controls. A small study examined interictal MEG in five adults with absence epilepsy and five matched controls [19]. Using coherence as the measure of interaction between channels, the authors found that average node strength, clustering coefficient, and global efficiency were all greater in patients than normal controls; these findings would be in keeping with ours. A group of 26 adults with IGE characterised by generalized tonic-clonic seizures was compared with 26 normal controls using fMRI and DTI [20]. The brain was parcellated into a large number of nodes, and connectivity between all pairs of nodes estimated from both datasets. The results were somewhat inconsistent between methods, but a decrease in small worldness and a decrease in clustering coefficient were found comparing patients with normals. A further study also used DTI to compare brain networks in 18 children with childhood absence epilepsy with 18 matched normal controls [21]. This study found that the network connection strength, clustering coefficient, local efficiency and global efficiency were decreased in the patients, and the characteristic path length increased. Although some of these findings are contradictory to our findings and those of [19], at the current time, it is extremely difficult to reconcile results found with MRI methods with those found using EEG/MEG.

Animal models of childhood absence epilepsy (CAE) show abnormalities in a complex brain network comprising a combination of a focal cortical region which drives the onset of generalised seizure discharges in thalamocortical networks, and an abnormality of anterior transcallosal pathways [22], [23]; this transcallosal abnormality has also been found in human juvenile myoclonic epilepsy (JME) [24], hence there is a justification to propose that large-scale brain network abnormalities are a feature of IGE. A large study of recent-onset IGE demonstrated 34–49% failed to achieve 12-month remission with first-line antiepileptic drugs [25], indicating an urgent need for better treatment based on improved mechanistic understanding of IGE. This improved understanding is likely to emerge from detailed phenotyping, genotyping, and the development of explanatory models. It seems likely that seizures emerge in large-scale brain networks through the interaction between brain network structure and the dynamics of the brain regions which constitute the network nodes [26]. We introduce the term “brain network ictogenicity” to describe the likelihood seizures will emerge from a brain network. In this study, we show that one contributor to brain network ictogenicity – network structure – is abnormal in IGE patients compared with healthy controls, and that a similar abnormality is observed in the unaffected relatives of the patients. We propose that our findings in the current study contribute to a more detailed phenotype of IGE and have implications for future genetic studies.

Fundamental to our approach is to identify a brain network endophenotype of IGE. An endophenotype is a heritable trait which is a component of a disorder or associated with high liability to develop the disorder. An endophenotype may be present in family members who do not have the disease, hence increasing the power of genetic studies, and its inheritance is likely to be simpler than the full disorder [6]. This concept has been extensively exploited in other common brain disorders with complex inheritance, such as schizophrenia [27]. Given the universal availability of EEG, and that GSW is a cardinal feature of IGE, EEG is an obvious place to look for an IGE endophenotype. It has been shown that 0.5% of unaffected adults and 1.8% of unaffected children under 16 yrs may show GSW [28], [29]. Unaffected first-degree relatives of patients with IGE show a much higher prevalence of GSW: 8–40% of unaffected siblings under 16 yrs had GSW when awake and up to 72% when asleep [30], [31]; but only 6–9% of unaffected siblings over 16 yrs had GSW [31], [32]. Therefore GSW may be an endophenotype of limited usefulness in adults, since, if IGE is explained by complex inheritance, at least 50% of first-degree relatives of patients with IGE should share one or more genes contributing to the IGE phenotype.

Conventional expert EEG review of our subjects revealed GSW in 49% of patients, 5% of relatives and zero controls; these findings are expected, and suggest that our cohort is unexceptional. Finding GSW in some “unaffected” relatives might suggest the possibility that some relatives in fact have unsuspected epilepsy. Although we concede this is possible, our detailed assessment of the relatives did not reveal any evidence of symptomatic seizures in any of the unaffected relatives group; post hoc exclusion of the two relatives with GSW does not alter the effects found.

Measures of EEG network topology differed between groups, revealing strong similarities between brain networks of patients and first degree relatives. For networks inferred from EEG band-pass filtered in the 6–9 Hz band, both the mean degree and mean degree variance was lower in normals than either patients or relatives. This indicates that the variability in the number of connections per network node is greater in patients and relatives, revealing the existence of a brain network endophenotype characterised by both unusually overconnected brain regions (hubs) and underconnected brain regions.

Comparison of epilepsy patients taking antiepileptic drugs with unmedicated normal controls introduces the potential confound that effects found may be due to the drugs and not due to the disease. We cannot exclude this possibility in our study. However, the relatives were unmedicated, therefore the comparison of relatives with controls does not suffer this confound.

Our network analyses were carried out in “sensor space” – that is, networks were constructed which described the interactions between activities at the EEG electrodes, rather than the interactions between the brain sources which generated these activities. The limited spatial sampling of routine clinical EEG would not readily permit source reconstruction, but future studies should attempt to identify the origins of these network properties in the brain.

We chose to examine weighted graphs, in contrast to some studies (eg. [33]) which have examined unweighted graphs. An unweighted graph is produced by choosing a threshold for edge weight, and assigning the value of an edge as either zero or one according to this threshold. As has been discussed in detail elsewhere [34], there are limitations to either approach. One practical limitation in our data is that our networks have only 19 nodes, therefore the range of possible network degree is limited; the consequence of this is that defining an unweighted network using a high threshold (or low network degree) would have the consequence that many networks will fall apart into disconnected components and therefore could not be validly compared; whereas using a low threshold (or high network degree) would have the outcome that the networks would tend to be fully connected (ie. every possible edge is present) therefore there would be very limited possibility to identify any difference between networks. Given these limitations, we argue that using a weighted unthresholded approach is preferable. Furthermore, some studies have compared between groups the weights of individual edges; we chose here to examine global properties, but have also examined for differences in individual edge strength finding no differences that survived Bonferroni correction.

There is an inherent problem in work of this kind, which may be described as the problem of reducing bias due to common sources of EEG activity seen at more than one scalp electrode, and which encompasses both the selection of reference electrode and consideration of the effect of volume conduction in selection of the method to determine interaction between EEG timeseries. The problem of common sources is well-known and does not have a single optimal solution [35] [36] [37]. We chose here to use an average reference, and to use a measure of interaction between EEG timeseries, PLF, which detects synchronization at zero phase lag. Note that previous work shows this combination of measure and reference is able to detect real differences in synchronization [37]; we are currently examining alternative measures of synchronization which may be less sensitive to volume conduction.

An important consideration in any experimental work is whether results are reliable and can be reproduced. An important strength of our study is the sample size: we have 117 subjects, and detected very large effect sizes, which is a strong defence against error. However, an important question is whether results are stable if a different epoch of EEG data were chosen from each subject. The difficulty of identifying artefact-free EEG data epochs of 20 s from every subject should not be underestimated – EEG is highly prone to movement, blink and other artefacts – and we chose to identify artefact-free epochs rather than clean the data using artefact removal tools. Hence, we were not able to find more than one suitable epoch for every subject. Nonetheless, post hoc, we sought to examine the stability of our findings by dividing the single epoch from each subject into two equal non-overlapping epochs of half the length (which we labelled epoch 1 and epoch 2). We repeated an identical analysis for both epochs from all subjects: in the analysis of the full 20 s epoch, we report five pairwise comparisons that reached significance using Bonferroni correction; using epoch 1 for every subject, the same 5 comparisons remained significant; using the epoch 2, three of the five comparisons remained significant and two were at the level of strong trend (and were significant without Bonferroni correction). Furthermore, the comparison between patients and relatives of mean degree, degree distribution variance, and clustering coefficient revealed no differences using the full 20 s epoch, and also revealed no differences using either epoch 1 or epoch 2. Therefore, our findings are reproducible within two non-overlapping epochs of EEG data. Nonetheless, we recognise that the reliability of our findings needs to be established in an independent dataset.

It is not yet established whether individual syndromes of IGE are entirely unrelated, with no shared aetiologic, genetic or mechanistic factors, or represent a continuum or set of overlapping disorders with important shared pathophysiology. We recognise in this context a divergence of views between those who seek to identify individual syndromes on the basis of highly detailed phenotyping, and those who seek common aetiological and mechanistic factors across the range of common IGE syndromes, as we do here. In this study, we specifically seek shared factors between families and between different IGE syndromes, hypothesising that there are likely to be shared genetic and mechanistic factors between different IGE syndromes [38] [39], [40]. We note this approach has been highly successful in recent genetic studies, which have identified recurrent chromosomal microdeletions as the most frequent identifiable genetic factor associated with all the common IGE syndromes studied here [41]–[43]. For example, the most frequently identified microdeletions each accounted for patients with at least three of the four common IGE syndromes included in our study here [42]: Microdeletions at 15q11.2 were identified in patients with JME, JAE, CAE and GTCS; microdeletions at16p13.11 were found in JME, CAE and GTCS; and microdeletions at 15q13.3 were found in JAE, JME and CAE. We argue that these genetic findings strongly support our argument that a similar brain network endophenotype might be found across the range of common IGE syndromes.

In summary, we show here for the first time the existence of a brain network endophenotype of IGE, present in relatives and patients. We propose that our findings have significant implications for the current mechanistic understanding of IGE, and for future phenotyping and genetics studies.
